# Validation of Serological Methods for COVID-19 and Retrospective Screening of Health Employees and Visitors to the São Paulo University Hospital, Brazil

**DOI:** 10.3389/fcimb.2022.787411

**Published:** 2022-06-02

**Authors:** Robert Andreata-Santos, Rafael Rahal Guaragna Machado, Rúbens Prince dos Santos Alves, Natiely Silva Sales, Camila Pereira Soares, Karine Bitencourt Rodrigues, Mariângela Oliveira Silva, Marianna Teixeira de Pinho Favaro, Mônica Josiane Rodrigues-Jesus, Márcio Massao Yamamoto, Juliana Bannwart de Andrade, Ricardo Ambrósio Fock, Paulo Francisco Ramos Margarido, Cristiane Rodrigues Guzzo Carvalho, Silvia Beatriz Boscardin, Edison Luiz Durigon, Luís C. S. Ferreira

**Affiliations:** ^1^ Vaccine Development Laboratory, Biomedical Sciences Institute, University of São Paulo, São Paulo, Brazil; ^2^ Retrovirology Laboratory, Immunology and Microbiology Department, Federal University of São Paulo, São Paulo, Brazil; ^3^ Clinical and Molecular Virology Laboratory, Microbiology Department, Institute of Biomedical Sciences, University of São Paulo, São Paulo, Brazil; ^4^ Department of Parasitology, Institute of Biomedical Sciences, University of São Paulo, São Paulo, Brazil; ^5^ School of Pharmaceutical Sciences, University of São Paulo, São Paulo, Brazil; ^6^ Clinical Laboratory Division, Pharmacy and Clinical Laboratory Department, University Hospital, University of São Paulo, São Paulo, Brazil; ^7^ Molecular and Structural Biology, Secretion Systems and c-di-GMP Signalling Laboratory, Department of Microbiology, Institute of Biomedical Sciences, University of São Paulo, São Paulo, Brazil; ^8^ Scientific Platform Pasteur/USP, University of São Paulo, São Paulo, Brazil

**Keywords:** SARS-CoV-2, serology, ELISA, health employees, ECLIA, surveillance

## Abstract

Reliable serological tests for the detection of SARS-CoV-2 antibodies among infected or vaccinated individuals are important for epidemiological and clinical studies. Low-cost approaches easily adaptable to high throughput screenings, such as Enzyme-Linked Immunosorbent Assays (ELISA) or electrochemiluminescence immunoassay (ECLIA), can be readily validated using different SARS-CoV-2 antigens. A total of 1,119 serum samples collected between March and July of 2020 from health employees and visitors to the University Hospital at the University of São Paulo were screened with the Elecsys^®^ Anti-SARS-CoV-2 immunoassay (Elecsys) (Roche Diagnostics) and three in-house ELISAs that are based on different antigens: the Nucleoprotein (N-ELISA), the Receptor Binding Domain (RBD-ELISA), and a portion of the S1 protein (ΔS1-ELISA). Virus neutralization test (CPE-VNT) was used as the gold standard to validate the serological assays. We observed high sensitivity and specificity values with the Elecsys (96.92% and 98.78%, respectively) and N-ELISA (93.94% and 94.40%, respectively), compared with RBD-ELISA (90.91% sensitivity and 88.80% specificity) and the ΔS1-ELISA (77.27% sensitivity and 76% specificity). The Elecsys^®^ proved to be a reliable SARS-CoV-2 serological test. Similarly, the recombinant SARS-CoV-2 N protein displayed good performance in the ELISA tests. The availability of reliable diagnostic tests is critical for the precise determination of infection rates, particularly in countries with high SARS-CoV-2 infection rates, such as Brazil. Collectively, our results indicate that the development and validation of new serological tests based on recombinant proteins may provide new alternatives for the SARS-CoV-2 diagnostic market.

## Introduction

The emergence of severe acute respiratory syndrome coronavirus 2 (SARS-CoV-2), the causative agent of Coronavirus disease 2019 (COVID-19), is responsible for the second pandemic of this century, according to the World Health Organization (WHO, 2020b), and a death toll that is still increasing. SARS-CoV-2 is an enveloped virus with positively oriented single-stranded RNA, and a ~30-kb genome that belongs to the *Coronaviridae* family (Grifoni et al., 2020). This virus causes a disease that is usually associated with asymptomatic manifestations that might progress to acute respiratory syndrome, which can lead to death (Xu et al., 2020). Approximately 20% of individuals with COVID-19 require hospitalization and exhibit flu-like symptoms, including fever, dry cough, and headache, that can progress to pneumonia, acute respiratory distress syndrome, septic shock, and cardiovascular manifestations (Guan et al., 2020; Mallah et al., 2021). The broad range of symptoms shared among other respiratory diseases contributed to the rapid spread of COVID-19 globally, reinforcing the need for accurate diagnostic tests for the disease. In this context, reliable serological tests for the detection of anti-SARS-CoV-2 AB in infected or vaccinated individuals are important for epidemiological and clinical studies.

Low-cost approaches that are easily adapted to high throughput screenings, such as Enzyme-Linked Immunosorbent Assays (ELISA) or electrochemiluminescence immunoassay (ECLIA), can be readily validated with different SARS-CoV-2 antigens. Among the most relevant antigen targets are the nucleoprotein (N) and spike (S) proteins. The S protein is essential for cell entry *via* the ACE-2 receptor. The high level of neutralizing antibody production against the S protein during natural infections, especially against the Receptor Binding Domain (RBD) (Sun et al., 2020; Achiron et al., 2021), demonstrates the potential of the S protein, or fragments derived from it, as a target antigen in serological tests. The N protein is another immunodominant antigen that is widely used for the serological detection of coronaviruses both in animals and humans (Leung et al., 2004; Abdelwahab et al., 2015). Nonetheless, among vaccines, antibodies targeting the S protein correlate with virus neutralization activity, thereby encouraging its use as a potential serological marker to differentiate vaccinated from infected individuals.

In the present study, we used serum samples previously tested for the presence of neutralizing antibodies produced after SARS-CoV-2 infection to validate a well-established ECLIA diagnosis test based on the N protein and three new ELISA strategies based on the N protein and different recombinant forms of the S protein. The tests with the best performance were applied to a cohort of health employees and visitors to the University Hospital of the University of São Paulo. Overall, our findings revealed a high correlation of ELISA results with the presence of neutralizing antibodies to SARS-CoV-2.

## Material and Methods

### Samples and Ethics

Human blood samples were obtained through venipuncture with vacutainers holding 5 mL tubes containing clot activator (Becton Dickinson). The tubes were stored at 4°C prior serum processing, which occurred at the same day by 30 min room temperature incubation followed by 10 min 805 *x g* centrifugation. Serum samples were separated from red blood cells (RBC) by pipetting, inactivated at 56°C for 30 min and stored at -20°C prior serological analysis.

All samples tested in this study were obtained after written consent. The study was approved under the CEPSH.007.2021 project number by the Human Research Ethics Committee of the Institute of Biomedical Sciences at the University of São Paulo.

### Protein Production and Purification

N-ELISA employs a solid-phase antigen corresponding to the complete N protein produced in a prokaryotic system and is commercially obtained (FAPON Biotech-China), while RBD-ELISA and ΔS1-ELISA use Spike (S) protein fragments as solid-phase antigens that are produced in-house. The plasmid encoding RBD was kindly provided by Dr. Florian Krammer (Icahn School of Medicine at Mount Sinai, USA). Of note, the protein was produced exactly as previously described (Stadlbauer et al., 2020). Briefly, RBD was expressed using the Expi293™ expression system (Thermo Scientific), as per the manufacturer’s recommendations. At the end of culture, the cell culture was centrifuged at 1,600 x g (EPPENDORF CENTRIFUGE 5810-R) for 10 min at room temperature, and the supernatant containing the recombinant protein was subjected to single-step nickel-based affinity chromatography in the presence of PBS-1x buffer at pH 7.4. For ΔS1, the BL21-RP strain was transformed by heat shock (Sambrook and Russell, 2001) with the expression vector encoding the spike fragment and cultivated in Terrific Broth (TB) medium supplemented with chloramphenicol (30 µg/ml) at 37°C on an orbital shaker (EPPENDORF – INNOVA S44i) set to 200 rpm until an Optical Density (OD_600nm_) of 2 was obtained. ΔS1 expression was induced with 0.5 mM Isopropyl β-D-1-thiogalactopyranoside (IPTG) for 18 h at 18°C. The resulting cell mass was lysed in a homogenizer (APLAB – ARTEPEÇAS) in the presence of Tris pH 9.0 buffer (0.1 M Tris, 0.2 M NaCl, 10% Glycerol). The insoluble extract was denatured in the presence of 6 M urea and submitted to a refolding process by pulsed dilution as previously described (Amorim et al., 2010). The refolded sample was subjected to single-step nickel-based affinity chromatography and eluted in the presence of 1 M of imidazole. Purifications were performed using the Akta Püre system (GE Healthcare) and the proteins obtained were quantified using a BSA curve (Bovine Serum Albumin) on SDS-PAGE (BIORAD – Universal Hood III).

### ELISAs

Specific IgG antibodies present in serum samples from each individual were qualitatively evaluated using ELISA according to a modified protocol based on a ZIKV NS1-based test previously reported (Kanno et al., 2020). Briefly, 96-well polystyrene COSTAR microplates (Corning Inc., New York, EUA) were coated with 200 ng of recombinant fragments encoding the whole SARS-CoV-2 N protein (N-ELISA) or the region 1 from the SARS-CoV-2 S protein (ΔS1-ELISA), both produced after *Escherichia coli*, as well as the RBD region produced by Expi293™ cells (RBD-ELISA) in a pH 9.6 carbonate/bicarbonate buffer. Blockage was performed *via* a 3 h incubation of the wells with PBS supplemented with lysine and mannitol. After the blocking agent was removed, sera samples diluted 1:100 in sample solution containing Tris-NaCl buffer supplemented with casein and EDTA were incubated in each well at 37°C for 60 min. The wells were washed three times in PBS-TWEEN 0.05% (PBST) solution and incubated with anti-human IgG conjugated to peroxidase (Sigma Aldrich™ Sigma, USA) at 37°C for 60 min. After a final wash, the wells were stained with Tetramethylbenzidine (Aldrich™ Sigma, USA). The reaction was stopped after 10 min by the addition of 100 µL of H_2_SO_4_ at 0.2 N. The OD reading was measured at 450 nm in a plate reader (Labsystems Multiscan, ThermoScientific, USA).

### Elecsys^®^ Anti-SARS-CoV-2 Immunoassay

The anti-SARS-CoV-2 Elecsys (Roche Diagnostics) is based on a double antigen ECLIA sandwich test that utilizes the N protein to detect specific SARS-CoV-2 antibodies (Muench et al., 2020). The tests were performed using an automated dosing system (Roche Diagnostics, Cobas^®^ e801 analytical unit) according to the manufacturer’s instructions. A signal to cut-off <1.0 for negative detection and ≥ 1.0 for positive detection were determined for interpretation of the results.

### Cytopathic Effect-Based Virus Neutralization Test (CPE-VNT)

For the neutralization assays, monolayers containing 5x10^4^ Vero cells (ATCC CCL-81) in 96-well culture plates were exposed to 1x10^3^ TCID_50_/mL of SARS-CoV-2/human/BRA/SP02/2020 strain (MT126808.1) previously incubated with 1:20, 1:40, and 1:80 of each evaluated sera, in a final volume of 150 µl. After a 3-day incubation, all wells were evaluated by optical microscopy for the presence of characteristic SARS-CoV-2 cytopathic effects, as previously described (Araujo et al., 2020; Wendel et al., 2020). The absence of cytopathic effects in at least the 1:20 dilution sample was considered a positive result of neutralizing antibodies to SARS-CoV-2. All procedures related to CPE-VNT were performed in a biosafety level 3 laboratory at the Institute of Biomedical Sciences, University of Sao Paulo, according to the WHO recommendations (WHO, 2020a).

### Statistical Analysis

All statistical analyses were performed, and figures were created using GraphPad Prism version 9.0.1, GraphPad Software (San Diego, CA, USA - www.graphpad.com). Receiver Operating Characteristic Curve (ROC curves) results were calculated according to (DeLong et al., 1988). The positive and negative samples according to the cut-off established as well as the likelihood ratio (LR), confidence intervals (CI), standard error (SE) and area under the curve (AUC) obtained by ROC curve analysis were used to compare test performances. CPE-VNT results served as the gold standard methodology. Furthermore, Cohen’s kappa coefficient (Kappa) was used to measure the inter-rater reliability to increase the overall confidence in the study’s accuracy.

## Results

### Similar Absolute Detections Obtained for the Study Cohort After Serologic Evaluation Using the CPE-VNT, ELISA, and ECLIA Technologies

The serological study was performed with a cohort comprising health employees and visitors to the University Hospital at the University of São Paulo (UH-USP). The study was carried out between March and July of 2020, during the first phase of the SARS-CoV-2 pandemic in Brazil. A total of 1,119 serum samples were initially screened with the Elecsys^®^ Anti-SARS-CoV-2 immunoassay (Elecsys) (Roche Diagnostics) (**Supplementary Figure 1**). To confirm the results and validate a SARS-CoV-2 serum panel, we selected previously positive (n=129), inconclusive (n=6), and randomly negative (n=247) samples to be evaluated by CPE-VNT for the detection of SARS-CoV-2 neutralizing antibodies. The final SARS-CoV-2 serum panel comprised 382 samples, with 132 positive and 250 negative samples (**Supplementary Figure 1**). The serum panel was subsequently used to validate *in house* ELISAs using different recombinant proteins as solid phase bound antigens. We tested three SARS-CoV-2 recombinant proteins in the ELISA protocols: the RBD of the S protein produced in human cells (RBD-ELISA), the whole N protein, and a fragment based on the S1 subdomain (ΔS1), which are both produced in bacterial (*E. coli*) cells.

The results obtained with the CPE-VNT-validated serum panel (132 positive and 250 negative samples) were similar to those using RBD-ELISA and N-ELISA, with 135 positives/247 negatives and 138 positives/244 negatives, respectively; however, a higher number of positive samples (176 positives/206 negatives) was detected with the ΔS1-ELISA (**Supplementary Figure 2**). From the 6 inconclusive samples detected by Elecsys^®^, 2 were found positive and 4 negative through CPE-VNT, RBD-ELISA and ΔS1-ELISA analysis, but not through N-ELISA, that showed 1 positive and 5 negative samples (**Supplementary Table 1**). Moreover, we observed discordant sample detection among the methodologies, especially between the ELISA tests (**Supplementary Figure 3**).

### N Protein Detection Using N-ELISA and Elecsys Has a Higher Correlation With Virus Neutralization Regardless of Eukaryotic or Prokaryotic Production

**Figure 1:**
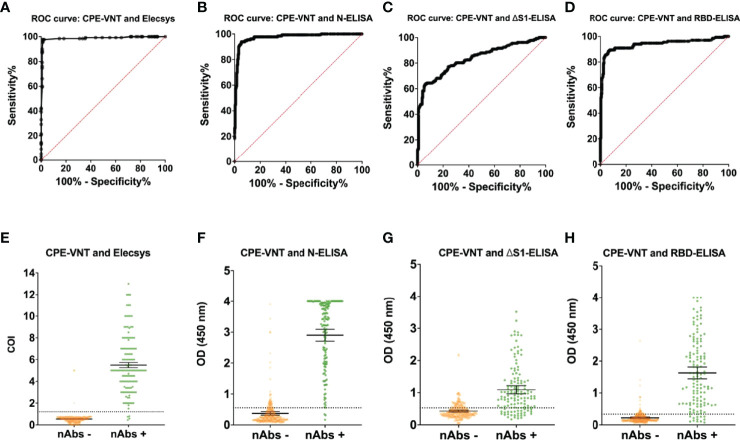
ROC curve analysis after CPE-VNT validation and data distribution of Elecsys^®^ Anti-SARS-CoV-2 Immunoassay and *in house* ELISA assays. The tests performances were calculated individually after CPE-VNT validation of the tested samples. All the performance analyses were obtained through ROC curves **(A–D)** and samples’ individual data **(E–H)** for the Elecsys^®^ Anti-SARS-CoV-2 Immunoassay **(A**, **E)**, N-ELISA assay **(B**, **F)**, ΔS1-ELISA assay **(C**, **G)** and RBD-ELISA assay **(D**, **H)**, respectively. Error bars and dashed lines represent 95% confidence interval (CI) and assay cut-off, respectively.

Further performance analyses of the evaluated ELISA were carried out using the CPE-VNT results as the gold standard. The sensitivity and specificity values were particularly high based on Elecsys (96.92% and 98.78%, respectively) and N-ELISA (93.94% and 94.40%, respectively) (**Table 1**). A reliable detection was obtained with the RBD-ELISA, with 90.91% sensitivity and 88.80% specificity. In contrast, the ΔS1-ELISA displayed 77.27% sensitivity and 76% specificity (**Table 1**). The Kappa values used to measure inter-rater reliability for the qualitative values evaluated followed the same pattern (**Table 1**), while the AUC from the ROC curves generated for each methodology (**Figures 1A–D**) showed minimal distinction between the Elecsys and N-ELISA (**Table 1**). Interestingly, although Elecsys and N-ELISA share the same antigen (N protein), a higher signal tendency was displayed by the N-ELISA positive samples, (**Figures 1E, F**). However, the RBD-ELISA and ΔS1-ELISA signal distribution appeared to follow the same pattern of Elecsys, with a higher number of samples around the median detection levels (**Figures 1E, G, H**).

**Table 1:** Performance of the Elecsys Anti-SARS-CoV-2 Immunoassay and *in house* ELISA assays after CPE-VNT validation.

	Elecsys anti-SARS-CoV-2 immunoassay^2^			N-ELISA^3^		ΔS1-ELISA^4^		RBD-ELISA^5^	
CPE-VNT^1^	Positive (AB≥ 1.0)	Negative (AB<1.0)	Inconclusive	Positive (OD^6^ ≥ 0.554)	Negative (OD <0.554)	Positive (OD ≥ 0.528)	Negative (OD <0.528)	Positive (OD ≥ 0.336)	Negative (OD <0.336)
**Positive (Titer ≥ 20)**	126	04	02	124	08	114	18	120	12
**Negative (Titer < 20)**	03	243	04	14	236	62	188	15	235
**Sensitivity [95% CI^7^]**	96.92% (126/130) [92.36%-98.80%]			93.94% (124/132) [88.50%-96.90%]		77.27% (114/132) [69.41%-83.59%]		90.91% (120/132) [84.78%-94.72%]	
**Specificity [95% CI]**	98.78% (243/246) [96.48%-99.67%]			94.40% (236/250) [90.82%-96.64%]		76.00% (188/250) [70.34%-80.88%]		88.80% (235/250) [84.29%-92.14%]	
**Kappa value ± SE^8^ [95% CI]**	0.959 ± 0.015 [0.929-0.989]			0.874 ± 0.026 [0.823-0.925]		0.571 ± 0.041 [0.490-0.652]		0.845 ± 0.029 [0.788-0.901]	
**Likelihood Ratio**	79.48			16.77		3.22		8.117	
**AUC ± SE [95% CI]**	0.9785 ± 0.009 [0.9596-0.9975]			0.9743 ± 0.008 [0.9586-0.9900]		0.8362 ± 0.023 [0.7903-0.8821]		0.9380 ± 0.016 [0.9063-0.9696]	

1 – Cytopathic effect-based virus neutralization test. Serum samples with reverse titers ≥ 20 were considered positive.

2 – For the Elecsys assay, a signal to cut-off <1.0 for negative detection and ≥ 1.0 for positive detection was determined for the results’ interpretation.

3 – For the N-ELISA assay, a signal to cut-off < 0.554 for negative detection and ≥ 0.554 for positive detection was determined for the results’ interpretation.

4 – For the ΔS1-ELISA, a signal to cut-off < 0.528 for negative detection and ≥ 0.528 for positive detection was determined for the results’ interpretation.

5 – For the RBD-ELISA, a signal to cut-off < 0.336 for negative detection and ≥ 0.336 for positive detection was determined for the results’ interpretation.

6- Optical Density (OD).

7 – Confidence Interval (CI).

8 – Standard Error (SE).

### Whole Cohort Analysis With N-ELISA and Elecsys Reveals Similar Detection and Prevalence

**Figure 2:**
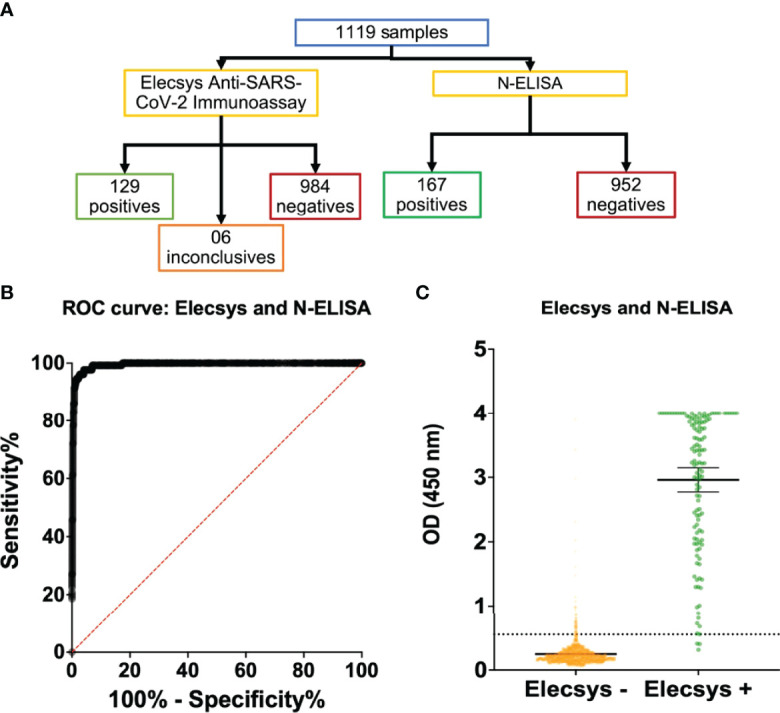
Global human sample evaluation with Elecsys^®^ Anti-SARS-CoV-2 Immunoassay and N-ELISA assay. The two assays that showed best performances were chosen to be evaluated by a global sample panel in the study. **(A)** Flow chart indicating the total evaluated samples and positive, inconclusive or negative results for Elecsys^®^ Anti-SARS-CoV-2 Immunoassay and N-ELISA assay. **(B, C)** ROC curve **(B)** and samples individual data **(C)** of N-ELISA assay performance with regard to Elecsys^®^ Anti-SARS-CoV-2 Immunoassay as gold standard test.

The serum samples of the whole cohort were monitored using Elecsys and N-ELISA. In these conditions, 129 positive, 6 inconclusive, and 984 negative samples were detected using the Elecsys assay while 167 positive and 952 negative samples were detected using N-ELISA (**Figure 2A**), which corresponded to 11.53% and 14.95% of positive seroconversion, respectively. When the Elecsys was regarded as a gold standard test for whole cohort analysis, only 1 positive sample detected by Elecsys was not detected by N-ELISA, while 40 Elecsys negative samples were considered positive. Such comparison resulted in an increase in the sensitivity (97.67%) and specificity (95.93%) of the N-ELISA (**Table 2**). Moreover, the N-ELISA’s Kappa value was slightly reduced while the AUC obtained after ROC curve calculation was enhanced (**Table 2** and **Figure 2B**). Similarly, the signal detection levels were significantly high and not evenly distributed around the median value when the results of N-ELISA and Elecsys were compared (**Figure 2C**). Taken together, our data validated the Elecsys^®^ Anti-SARS-CoV-2 immunoassay as a reliable SARS-CoV-2 serological test and revealed the good performance of an ELISA based on the recombinant SARS-CoV-2 N protein.

**Table 2:** N-ELISA performance with regard to the Elecsys Anti-SARS-CoV-2 Immunoassay as gold standard test.

N-ELISA	Elecsys Anti-SARS-CoV-2 Immunoassay^1^	
	Positive (COI^2^ ≥ 1.2)	Negative (COI < 0.8)
**Positive (OD^3^ ≥ 0.554)**	128	40
**Negative (OD < 0.554)**	01	944
**Sensitivity [95% CI]**	97.67% (128/129) [93.39%-99.37%]	
**Specificity [95% CI]**	95.93% (944/984) [94.51%-97.0%]	
**Kappa value ± SE [95% CI]**	0.841 ± 0.024 [0.794-0.888]	
**AUC ± SE [95% CI]**	0.9942 ± 0.0019 [0.9904-0.9981]	

1 – For the Elecsys assay, a signal to cut-off <1.0 for negative detection and ≥ 1.0 for positive detection was determined for the results’ interpretation.

2 – Cut-off Index (COI).

3- Optical Density (OD).

## Discussion

The validation of presently available tests and the development of new SARS-CoV-2 serodiagnosis tests are relevant for tracking infection and vaccination rates during the COVID-19 pandemic. Here, we validated a well-established diagnosis test commonly used as reference at diagnostic centers and evaluated three new in-house ELISA strategies. Positive Elecsys results were found to highly correlate with the presence of neutralizing antibodies to SARS-CoV-2. Similar results were also observed with the ELISA based on the N protein produced by prokaryotic cells (N-ELISA). When the two methodologies were compared, slightly better detection and specificity were observed with the Elecsys test while higher detection signals were observed with the N-ELISA. Notably, the two tests rely on the use of the N protein while positive CPE-VNT is a measure of the presence of antibodies against structural proteins, particularly the S protein. Results compatible with a reliable diagnostic use were also obtained with the RBD-ELISA, which is based on a recombinant protein produced in eukaryotic cells. The overall statistical parameters evaluated in the establishment of these tests revealed reliable diagnostic results and a high probability of accurate positive and negative detections. Furthermore, the evaluation of the tested cohort presented similar prevalence numbers obtained with the two tests with better performances. Therefore, the present results endorse the use of the evaluated tests and concomitantly enabled the validation of two *in house* ELISA approaches.

The current availability of SARS-CoV-2 tests is still limited despite the frequent increase in cases in different regions worldwide. This is especially true in countries, such as Brazil, where the public health system is on the verge of collapsing. Thus, the development of new tests with increased cost benefit and based on technologies commonly available in laboratories and hospitals that permit rapid implementation is of great importance. In this scenario, conventional ELISA represents a technology that is more available than bioluminescence tests, such as the Elecsys assay. Nonetheless, bioluminescence tests, such as the Elecsys, are better suited for high throughput screenings performed at reference laboratories.

Recombinant proteins are commonly employed as antigens for detection in diagnostic tests (Cuzzubbo et al., 2001; Balamurugan et al., 2010). Due to its low-cost and rapid production, fragments or whole proteins can be successfully used as the basis for the development of specific serology tests. Among the different platforms available for recombinant protein production, those with the best cost/benefit ratio are based on the use of prokaryotic cells, particularly those based on *E. coli*. Despite a lack of glycosylation, proteins produced by prokaryotic cells are used in most commercially available COVID-19 serological tests, maintaining high sensitivity and specificity levels (Rosati et al., 2003; Yathi et al., 2011). In fact, the results from N-ELISA were equivalent to those of the Elecsys assay, which is based on the N protein produced in eukaryotic cells, thereby confirming the usefulness of antigens produced in bacterial cells for the development of COVID-19 serological tests.

Viral surface-exposed proteins produced in eukaryotic cells may display better diagnostic performance in serological tests for antigens produced in bacterial cells. Indeed, our results support previous observations that the detection of antibodies targeting surface-exposed proteins is improved using glycosylated antigens (Brigger et al., 2021; Zhang et al., 2021). In the present study, this finding was confirmed using ΔS1-ELISA and RBD-ELISA. In this regard, the use of recombinant proteins produced in different technological platforms for the serological screening of SARS-CoV-2-infected or vaccinated people should consider performance and costs.

The serological tests evaluated in the present study achieved excellent performance, with similar or even superior performance to that of other available SARS-CoV-2 serology kits (Deeks et al., 2020; Kohmer et al., 2020; Mendrone‐Junior et al., 2021). The whole cohort prevalence results showed higher seroconversion than previously reported for 133 sentinel cities in all Brazilian states (Hallal et al., 2020); this might be due to the samples from this study being exclusively obtained in the São Paulo state, which had most of the SARS-CoV-2 cases reported in Brazil. Furthermore, the São Paulo University Hospital acted as a Long-Term Care Facility during the Covid-19 pandemic. Such facilities reported similar seroprevalence in Brazil after a post-outbreak setting (De Barros et al., 2021). In fact, our results are similar or inferior to most international observations from the same evaluation period (Mosites et al., 2020; Hobbs et al., 2021; Mulenga et al., 2021), where several countries had similar attack rates owing to SARS-CoV-2 infection. Collectively, the present work indicates that the development and validation of new serological tests based on recombinant proteins may offer new and reliable alternatives for the SARS-CoV-2 diagnostic market.

## Data Availability Statement

The raw data supporting the conclusions of this article will be made available by the authors, without undue reservation.

## Ethics Statement

The study was approved under the CEPSH.007.2021 project number by the Human Research Ethics Committee of the Institute of Biomedical Sciences at the University of São Paulo. The patients/participants provided their written informed consent to participate in this study.

## Author Contributions

RA-S, study design, ELISA data collection, data analysis, figure construction, and writing. RM, study design, VNT data collection, data analysis, figure construction, and writing. RA, NS, KR, and MS, ELISA data collection and writing. NS, ELISA data collection and writing. CS, VNT data collection and writing. MF and MJ, ΔS1 recombinant protein production and writing. MY, RBD recombinant protein production and writing. JA, sample collection and Elecsys data collection. RF and PM, sample collection, Elecsys data collection, and writing. CC, ΔS1 recombinant protein production. SB, study design, RBD recombinant protein production, and writing. ED, structure, study design, data analysis, figure construction, and writing. LF, guarantor, structure, study design, data analysis, figure construction, and writing. All authors contributed to the article and approved the submitted version.

## Funding

This work was supported by Fundação de Amparo à Pesquisa do Estado de São Paulo (FAPESP): [thematic project No. 2016/20045-7 (LF)], [PhD scholarship No. 2016/23560-0, Postdoc scholarship 2021/05661-1 from project No. 2020/08943-5 (RA-S)], [grants No. 2018/07142-9 and 2014/50890-5 (SB)], [projects No. 2017/24769-2 (RM)], [2018/23680-0 (CS)], [2016/20045-7, 2020/06409-1 (ED)], [2020/10700-3 (MF)], [2018/07629-5 (MS)] and [2016/14344-1 (NS)]. This work was supported by Fundação de Amparo à Pesquisa do Estado de São Paulo (FAPESP)/Coordenação de Aperfeiçoamento de Pessoal de Nível Superior (CAPES): [No. 2015/02352-7 (RA)]. This work was supported by Coordenação de Aperfeiçoamento de Pessoal de Nível Superior (CAPES): [No. 88887.467980/2019-00 (KR)], [88887.185337/2018-00 (MJ)]. This work was supported by Conselho Nacional de Desenvolvimento Científico e Tecnológico (CNPq) [No. 401506/2020-7].

## Conflict of Interest

The authors declare that the research was conducted in the absence of any commercial or financial relationships that could be construed as a potential conflict of interest.

## Publisher’s Note

All claims expressed in this article are solely those of the authors and do not necessarily represent those of their affiliated organizations, or those of the publisher, the editors and the reviewers. Any product that may be evaluated in this article, or claim that may be made by its manufacturer, is not guaranteed or endorsed by the publisher.
