# Supplementary material for: Validation of Serological Methods for COVID-19 and Retrospective Screening of Health Employees and Visitors to the São Paulo University Hospital, Brazil
Source: Front Cell Infect Microbiol. 2022 Jun 2;12:787411. doi: 10.3389/fcimb.2022.787411 (PMC9202673; doi:10.3389/fcimb.2022.787411)
Supplement: Supplementary file 1 [file DataSheet_1.docx]

**Supplementary Figures.**


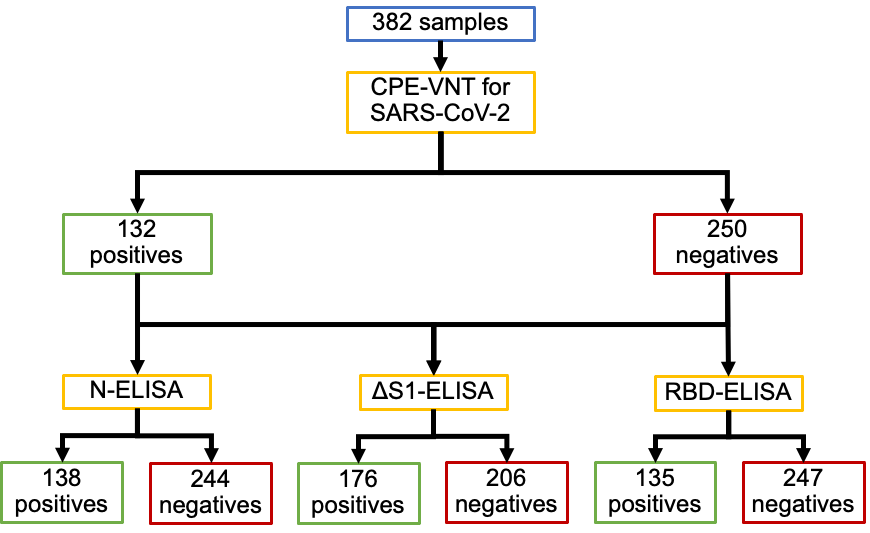


**Figure 1S. CPE-VNT validation and *in house* evaluation of the study selected samples.** The 382 samples previously selected for validation were evaluated by CPE-VNT regarding neutralizing antibodies and subsequently tested by three *in house* ELISA methodologies (N-ELISA, ΔS1-ELISA and RBD-ELISA) for detection of SARS-CoV-2 specific antibodies. The figure shows a flow chart indicating CPE-VNT and ELISA positive and negative samples.


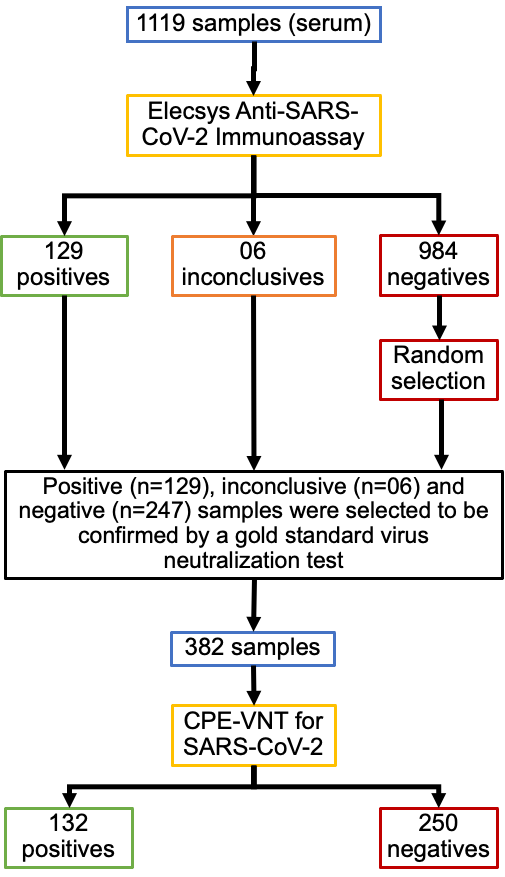


**Figure 2S. Flow chart representing sample selection for the study.** Elecsys^®^ Anti-SARS-CoV-2 Immunoassay evaluated samples were selected for the study based on positive, inconclusive or negative results.


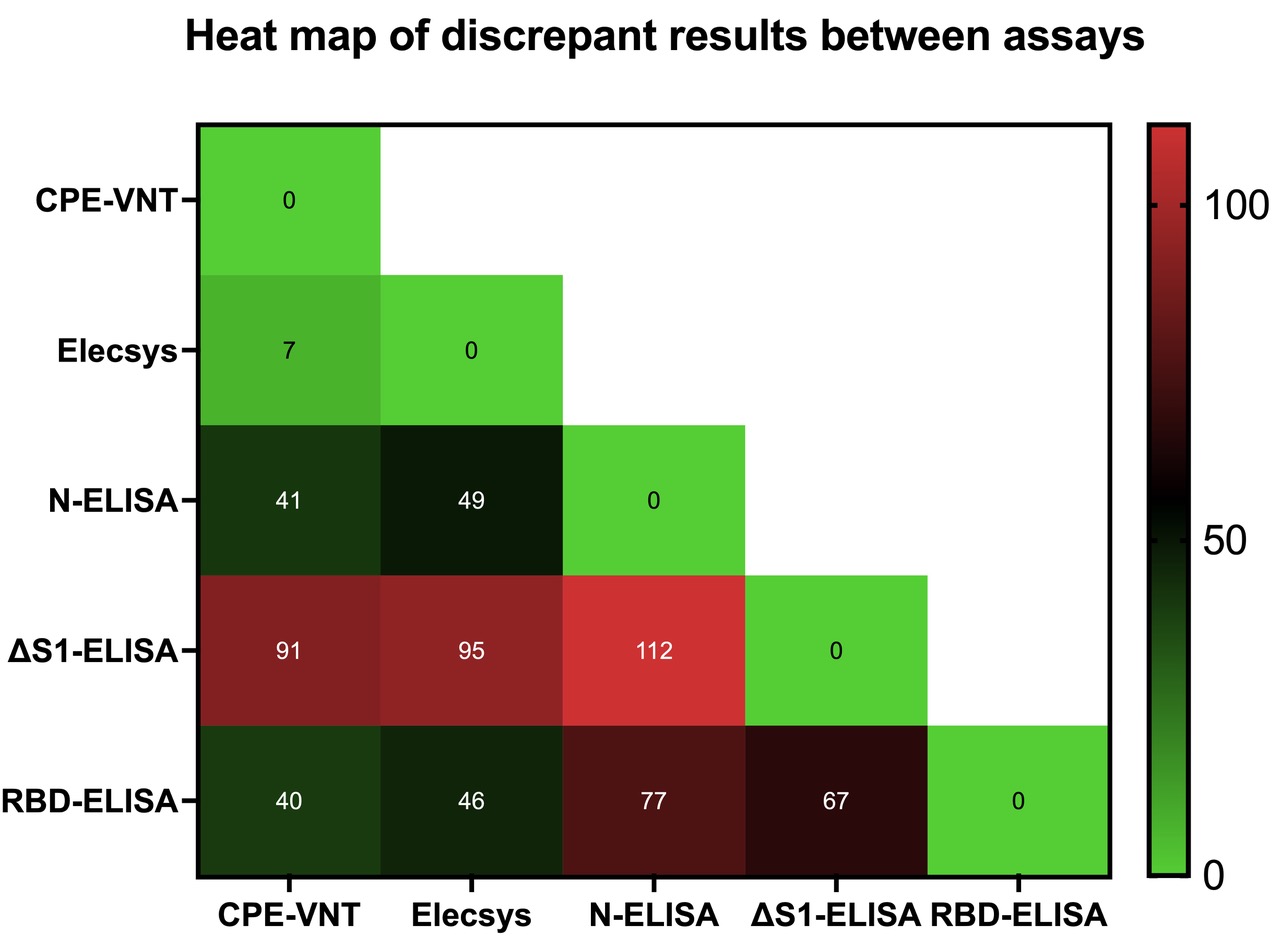


**Figure 3S. Heat map of discrepant results between assays.** The heat map represents, from light green to red colors, the respective crescent number of samples that had different results when evaluated by the assays of the study.

**Supplementary Table.**

**Table S1. Inconclusive Elecsys results for CPE-VNT, N-ELISA, ΔS1-ELISA and RBD-ELISA tests.**

| **Sample ID** | **Elecsys anti-SARS-CoV-2 immunoassay** | **CPE-VNT**  ***** | **N-ELISA**  ****** | **ΔS1-ELISA**  ******* | **RBD-ELISA**  ******** |
| --- | --- | --- | --- | --- | --- |
| 0886625101 | Inconclusive | **-** | **-** | **+** | **-** |
| 0886574301 | Inconclusive | **-** | **-** | **-** | **-** |
| 0886667501 | Inconclusive | **+** | **-** | **+** | **+** |
| 0886677001 | Inconclusive | **-** | **-** | **-** | **-** |
| 0886630401 | Inconclusive | **-** | **-** | **-** | **-** |
| 0886618801 | Inconclusive | **+** | **+** | **+** | **+** |

* Positive (+) = VNT_100_ ≥ 20; Negative (-) = VNT_100_ < 20

** (+) = OD ≥ 0.554; (-) = OD < 0.554

*** (+) = OD ≥ 0.528; (-) = OD < 0.528

**** (+) = OD ≥ 0.336; (-) = OD < 0.336
